# Tópicos Emergentes em Insuficiência Cardíaca: O Futuro na Insuficiência Cardíaca: Telemonitoramento, *Wearables* , Inteligência Artificial e Ensino na Era Pós-Pandemia

**DOI:** 10.36660/abc.20201127

**Published:** 2020-12-01

**Authors:** Aguinaldo F Freitas, Fábio S. Silveira, Germano E. Conceição-Souza, Manoel F. Canesin, Pedro V. Schwartzmann, Sabrina Bernardez-Pereira, Reinaldo B. Bestetti

**Affiliations:** 1 Hospital das Clínicas Universidade Federal de Goiás GoiâniaGO Brasil Hospital das Clínicas da Universidade Federal de Goiás (HC-UFG), Goiânia, GO - Brasil; 2 Fundação Beneficência Hospital de Cirurgia AracajuSE Brasil Fundação Beneficência Hospital de Cirurgia (FBHC-Ebserh), Aracaju, SE - Brasil; 3 Centro de Pesquisa Clínica do Coração AracajuSE Brasil Centro de Pesquisa Clínica do Coração, Aracaju, SE - Brasil; 4 Hospital Alemão Oswaldo Cruz São PauloSP Brasil Hospital Alemão Oswaldo Cruz, São Paulo, SP - Brasil; 5 Hospital Regional de São José dos Campos São José dos CamposSP Brasil Hospital Regional de São José dos Campos, São José dos Campos, SP - Brasil; 6 Hospital Universitário Universidade Estadual de Londrina LondrinaPR Brasil Hospital Universitário - Universidade Estadual de Londrina (HU-UEL), Londrina, PR - Brasil; 7 ACTIVE - Metodologias Ativas de Ensino São PauloSP Brasil ACTIVE - Metodologias Ativas de Ensino, São Paulo, SP - Brasil; 8 Hospital Unimed Ribeirão Preto Ribeirão PretoSP Brasil Hospital Unimed Ribeirão Preto, Ribeirão Preto, SP - Brasil; 9 Centro Avançado de Pesquisa, Ensino e Diagnóstico Ribeirão PretoSP Brasil Centro Avançado de Pesquisa, Ensino e Diagnóstico (Caped), Ribeirão Preto, SP - Brasil; 10 Hospital de Coração São PauloSP Brasil Hospital de Coração (HCor), São Paulo, SP - Brasil; 11 Departamento de Medicina Universidade de Ribeirão Preto Ribeirão PretoSP Brasil Departamento de Medicina, Universidade de Ribeirão Preto (Unaerp), Ribeirão Preto, SP – Brasil

**Keywords:** Telemonitoramento, Inteligência Artificial, Insuficiência Cardíaca, Ensino

## Telemonitoramento na Insuficiência Cardíaca (IC) e Manejo Remoto

O telemonitoramento consiste no monitoramento e suporte à distância (TMO) do paciente com IC crônica. O TMO pode ser não invasivo (TMONI) ou invasivo (TMOI). O TMONI compreende ligações telefônicas, orientações periódicas por meio de material instrutivo, controle e monitoramento do peso corporal, vídeo – chamadas e teleconsultas.^1 , 2^ O TMOI envolve uma série de dispositivos implantáveis que transmitem, a um servidor à distância, informações hemodinâmicas e impedância intratorácica.^3 , 4^

Ensaios clínicos de TMONI costumam ter resultados conflitantes. No entanto, metanálises envolvendo estudos observacionais e randomizados de TMOI e TMONI têm mostrado impacto positivo no prognóstico de pacientes com IC. A redução na mortalidade geral pode variar de 19 a 31% com o TMOI ou TMIONI em pacientes com IC,^5 , 6^ enquanto a redução na frequência de internação hospitalar por IC varia de 27 a 39%, principalmente em pacientes em classe funcional III/IV.^7 - 9^

O TMOI com evidências mais convincentes na IC é o CardioMEMS,^7^ um dispositivo implantado via percutânea na artéria pulmonar, que transmite os valores pressóricos centrais para um servidor seguro e orienta o ajuste nas doses de diuréticos e vasodilatadores.

## *Wearables* na IC: Ferramentas de Monitoramento ou Gadget Eletrônico?

*Wearables* são ferramentas computacionais “vestíveis”. Podem ser um relógio, uma camisa, uma lente de contato ou um sapato, por exemplo. Esses aparelhos contêm sensores que podem obter dados em tempo real e transmiti-los para uma nuvem ou para outro dispositivo, permitindo análises de uma enorme quantidade de dados, bem como a sua disponibilização para decisões diagnósticas e terapêuticas. Tudo isso é permitido graças à evolução da tecnologia de transmissão de dados, com o advento do 5G.

Assim, a Internet das coisas (IoT) realmente se tornará progressivamente uma realidade em diversos países. Com a Internet das coisas médicas (IoMT), não será diferente. Com o progressivo barateamento dessas tecnologias, vencendo-se a barreira do custo-efetividade, teremos a oportunidade de testar uma infinidade de *wearables* que poderão permitir o acesso precoce da equipe de saúde a dados de telemonitoramento de variáveis como pressão arterial, pulso, saturação de oxigênio, análise postural, queda, frequência respiratória, temperatura, glicemia capilar, entre outros.

Isso poderá impactar em desfechos clinicamente relevantes, como hospitalizações, custos diretos e indiretos e até mortalidade, ao mesmo tempo em que se poderá direcionar o manejo dos pacientes com IC para uma medicina de precisão mais personalizada – um novo paradigma. Indubitavelmente, há a necessidade de validação de cada *gadget* que se proponha a trazer estes benefícios, levando em consideração as principais barreiras para sua implementação, mas definitivamente os *wearables* chegaram para ficar.^8 , 9^

## Inteligência Artificial e *Big Data* na IC

Sistemas computacionais capazes de realizar tarefas que originalmente exigiam atuação humana constituem a base da inteligência artificial (IA). Esses sistemas surgiram pela necessidade de interpretação de grande volume de dados ( *big data* ). Os sistemas devem ser capazes de analisar dados simples ou complexos, de forma veloz e com acurácia, além de se adaptarem a eles, sem uma programação estática.^10^ *Machine learning* (ML) e *deep learning* (DL) são extensões da IA. O ML é a prática de usar algoritmos para coletar dados, aprender com eles e então fazer a determinação ou predição de alguma coisa, de pessoas ou mesmo de pacientes. Para ser útil e fidedigno, precisa ser constantemente alimentado por dados reais. Além disso, o DL, que é o extremo da interação e adaptação de aprendizado, pode estabelecer conexões neurais e diversidade para integrar diferentes bases de dados.^11^

A IA, a ML e o DL já apresentam aplicações em estudo para IC, seja como diagnóstico, avaliação de prognóstico, TMO ou ainda para selecionar pacientes com maior benefício para diversas terapias. Isso pode ser feito, por exemplo, na distinção de fenótipos, alocando pacientes em diferentes perfis de assinatura de doença;^12^ na melhor acurácia para o diagnóstico de IC aguda em relação ao médico;^13^ e no eventual direcionamento para terapias novas ou já estabelecidas, como análise adicional do ECG basal para identificar paciente melhor respondedor à TRC.^14^

## Ensino Médico em IC nos Novos Tempos

Um dos grandes desafios dos estudos e pesquisa clínica é a sua capacidade de traduzi seus resultados científicos para a prática clínica. São vários os fatores envolvidos nesse processo; um importante fator é, particularmente, a capacidade de transmissão e aplicação efetiva em menor intervalo de tempo desse conhecimento para um maior número de profissionais.

As mudanças em ensino médico e, consequentemente, no tema IC, passam por uma revolução.^15^ O que há pouco tempo se resumia quase que exclusivamente a aulas expositivas, há anos, através de técnicas de metodologia ativa de ensino e mais recentemente por modelos híbridos sincrônicos ou assíncronos digitais, o ensino é aperfeiçoado, aprimorando o processo de aprendizagem e, consequentemente, o cuidado com o paciente.^16^

O modelo atual de ensino deve oferecer o conceito de andragogia,^17^ associado ao modelo de ensino AGES. Esse modelo engloba a construção de processos expositivos de aprendizagem curtos, que mantêm a atenção do expectador ou de alunos, associado à capacidade de geração de motivos intrínsecos que tragam significado na aprendizagem. Ou seja, envolve emoções que possam fortalecer o aprendizado, de forma que este seja construído em várias etapas ( *Spaced* ). Quando incorporados, todos estes pontos formam uma aprendizagem mais profunda e efetiva, levando ao médico e ao profissional de saúde a possibilidade de adquirir e aplicar na prática os cuidados em IC.^18^

## Lista de Participantes do Heart Failure Summit Brazil 2020 / Departamento de Insuficiência Cardíaca - DEIC/SBC

Aguinaldo Freitas Junior, Andréia Biolo, Antonio Carlos Pereira Barretto, Antônio Lagoeiro Jorge, Bruno Biselli, Carlos Eduardo Montenegro, Denilson Campos de Albuquerque, Dirceu Rodrigues de Almeida, Edimar Alcides Bocchi, Edval Gomes dos Santos Júnior, Estêvão Lanna Figueiredo, Evandro Tinoco Mesquita, Fabiana G. Marcondes-Braga, Fábio Fernandes, Fabio Serra Silveira, Felix José Alvarez Ramires, Fernando Atik, Fernando Bacal, Flávio de Souza Brito, Germano Emilio Conceição Souza, Gustavo Calado de Aguiar Ribeiro, Humberto Villacorta Jr., Jefferson Luis Vieira, João David de Souza Neto, João Manoel Rossi Neto, José Albuquerque de Figueiredo Neto, Lídia Ana Zytynski Moura, Livia Adams Goldraich, Luís Beck-da- Silva, Luís Eduardo Paim Rohde, Luiz Claudio Danzmann, Manoel Fernandes Canesin, Marcelo Bittencourt, Marcelo Westerlund Montera, Marcely Gimenes Bonatto, Marcus Vinicius Simões, Maria da Consolação Vieira Moreira, Miguel Morita Fernandes da Silva, Monica Samuel Avila, Mucio Tavares de Oliveira Junior, Nadine Clausell, Odilson Marcos Silvestre, Otavio Rizzi Coelho Filho, Pedro Vellosa Schwartzmann, Reinaldo Bulgarelli Bestetti, Ricardo Mourilhe Rocha, Sabrina Bernadez Pereira, Salvador Rassi, Sandrigo Mangini, Silvia Marinho Martins, Silvia Moreira Ayub Ferreira, Victor Sarli Issa.
