# Supplementary material for: Tópicos Emergentes em Insuficiência Cardíaca: O Futuro na Insuficiência Cardíaca: Telemonitoramento, Wearables , Inteligência Artificial e Ensino na Era Pós-Pandemia
Source: Arq Bras Cardiol. 2020 Dec 1;115(6):1190–2. [Article in Portuguese] doi: 10.36660/abc.20201127 (PMC8133716; doi:10.36660/abc.20201127)
Supplement: Translation

# Translation: Emerging Topics in Heart Failure: The Future of Heart Failure: Telemonitoring, Wearables, Artificial Intelligence and Learning in the Post-Pandemic Era

**Authors:** Aguinaldo F Freitas, Fábio S. Silveira, Germano E. Conceição-Souza, Manoel F. Canesin, Pedro V. Schwartzmann, Sabrina Bernardez-Pereira, Reinaldo B. Bestetti

**Affiliations:** 1 Brazil Hospital das Clínicas da Universidade Federal de Goiás (HC-UFG), Goiânia, GO - Brazil; 2 Brazil Fundação Beneficência Hospital de Cirurgia (FBHC-Ebserh), Aracaju, SE - Brazil; 3 Brazil Centro de Pesquisa Clínica do Coração, Aracaju, SE - Brazil; 4 Brazil Hospital Alemão Oswaldo Cruz, São Paulo, SP - Brazil; 5 Brazil Hospital Regional de São José dos Campos, São José dos Campos, SP - Brazil; 6 Brazil Hospital Universitário - Universidade Estadual de Londrina (HU-UEL), Londrina, PR - Brazil; 7 Brazil ACTIVE - Metodologias Ativas de Ensino, São Paulo, SP - Brazil; 8 Brazil Hospital Unimed Ribeirão Preto, Ribeirão Preto, SP - Brazil; 9 Brazil Centro Avançado de Pesquisa, Ensino e Diagnóstico (Caped), Ribeirão Preto, SP - Brazil; 10 Brazil Hospital de Coração (HCor), São Paulo, SP - Brazil; 11 Brazil Departamento de Medicina, Universidade de Ribeirão Preto (Unaerp), Ribeirão Preto, SP – Brazil

**Keywords:** Telemonitoring, Artificial Intelligence, Heart Failure, Learning

## Telemonitoring in Heart Failure and Remote Management

Telemonitoring consists of monitoring and remote support for chronic heart failure (HF) patients. Telemonitoring can be non-invasive or invasive. Non-invasive telemonitoring includes telephone calls, periodic guidance through instructional material, control and monitoring of body weight, video calls and teleconsultations.^1 , 2^ Invasive telemonitoring involves implantable devices that transmit hemodynamic and intrathoracic impedance data to a remote server.^3 , 4^

Clinical trials of non-invasive telemonitoring often show conflicting results. However, meta-analyses of observational and randomized studies on invasive and non-invasive telemonitoring have shown that they have a positive impact on HF prognosis. The reduction in overall mortality in HF patients can vary from 19 to 31% with invasive or non-invasive telemonitoring,^5 , 6^ while reduction in the frequency of hospitalization for HF varies from 27 to 39%, mainly in functional class III/IV patients.^7 - 9^

Among invasive telemonitoring systems, CardioMEMS^7^ has the most convincing evidence in HF. This device is implanted percutaneously in the pulmonary artery and transmits central blood pressure values to a secure server, guiding adjustments in diuretic and vasodilator dosage.

## Wearables in HF: Monitoring Tools or More Electronic Gadgets?

Wearables are computational tools that can be worn on the body. They could be a watch, a shirt, a contact lens, or a shoe, for example. These devices contain sensors that obtain real-time data and transmit it to a cloud or another device, allowing analysis of an enormous amount of data, as well as facilitating diagnostic and therapeutic decision making. All of this has been made possible by the evolution of data transmission technology, particularly the advent of 5G networks.

Thus, the Internet of things will eventually become a reality in a number of countries. The Internet of medical things will be no different. The progressive cheapening of these technologies will overcome the cost-effectiveness barrier, and we will have the opportunity to test a multitude of wearables that can provide the health team with early access to telemonitoring data for variables such as blood pressure, pulse, blood oxygen, postural analysis, fall, respiratory rate, temperature, capillary blood glucose, etc.

This could have an impact on clinically relevant outcomes, such as hospitalizations, direct and indirect costs and even mortality. At the same time, the management of HF patients can be directed towards more personalized precision medicine - a new paradigm. Despite the fact that each gadget promising such benefits must undoubtedly be validated (considering the main barriers to its implementation), it seems clear that wearables are here to stay.^8 , 9^

## Artificial Intelligence and Big Data in HF

Computer systems capable of carrying out tasks that originally required human performance are the basis of artificial intelligence. These systems were developed out of the need to interpret ‘big data’. Systems must be able to quickly and accurately analyze simple or complex data, as well as adapt to the data without static programming.^10^ Machine learning and deep learning are extensions of artificial intelligence. Machine learning uses algorithms to collect data, learn from them and then make predictions about things or even patients. To be useful and reliable, machine learning systems must be constantly fed real data. In addition, deep learning, which is the vanguard of interaction and adaptive learning, can involve neural connections and diversity to integrate different databases.^11^

Artificial intelligence, machine learning and deep learning applications are already being studied for HF with respect to diagnosis, prognosis assessment, telemonitoring or even the selection of patients with the greatest projected benefit from various therapies. This can occur, for example, by distinguishing phenotypes, allocating patients with different disease profiles;^12^ by determining the best acute HF diagnostic accuracy in relation to the doctor;^13^ and by targeting new or already established therapies, such as additional analysis of baseline electrocardiograms to identify the best patients for cardiac-resynchronization therapy.^14^

## Medical Education About HF in These New Times

One of the greatest challenges for studies and clinical research is translating scientific results into clinical practice. Although there are several factors involved in this process, one particularly important factor is the ability to effectively transmit and apply knowledge to the greatest number of professionals in the least possible time.

Medical education and, consequently, the subject of HF, are undergoing a revolution.^15^ For a long time, HF had been reduced almost exclusively to expository classes. However, in recent years, active teaching methodologies and synchronous hybrid models or digital asynchronous models have improved the teaching and learning process and, consequently, patient care.^16^

The current teaching model must offer the concept of andragogy,^17^ which is associated with the AGES teaching model. This model involves short expository learning processes, which maintain viewer/student attention, and produces intrinsic motives that bring meaning to learning. That is, it involves emotions that can strengthen learning, so that learning is spaced over several stages. When incorporated, these approaches lead to more profound and effective learning, enabling doctors and health professionals to absorb and apply the best HF care practices.
